# Appendicitis in an incisional hernia sac following renal transplantation: A case report and brief review of the literature

**DOI:** 10.1016/j.radcr.2021.04.026

**Published:** 2021-05-01

**Authors:** Dhairya A. Lakhani, Jafar Dada, Aneri B. Balar, Ahsan U. Khan, Zalak Patel, Brian Markovich, Thuan-Phuong Nguyen

**Affiliations:** a Department of Radiology, School of Medicine, West Virginia University, Morgantown, WV; b Section of Abdominal Radiology, Department of Radiology, School of Medicine, West Virginia University, Morgantown, WV; c Section of Musculoskeletal Radiology, Department of Radiology, School of Medicine, West Virginia University, 1 Medical Center Drive, Morgantown, WV 26506, USA

**Keywords:** Appendicitis, Appendiceal hernia, Incisional hernia

## Abstract

Acute appendicitis is a surgical emergency. However, the presence of vermiform appendix in a hernial sac is rare. It is even rarer to find inflamed appendix in an hernial sac. The most common site is right groin hernia (Inguinal > Femoral). There is low incidence of an incisional hernia following renal transplantation, as compared to patients with laparotomy. Appendicitis in hernial sac masquerades clinical presentation of an incarcerated hernia. Computed tomography plays a pivotal role in early diagnosis, demonstrating a dilated appendix with wall thickening and peri-appendiceal fat stranding. Patients are managed with appendectomy. The management of appendiceal hernias without inflammation remains controversial, with few reported cases managed with hernia sac repair or appendectomy. In this report were described a case of appendicitis in an incisional hernia following renal transplantation which was managed with appendectomy.

## Background

Acute appendicitis defined as inflammation of the appendix, have an annual incidence of 1 per 1000 person [1]. It rare to find inflamed appendix in the hernial sac [2,3]. The overall incidence of appendicitis occurring in hernias (including femoral, inguinal or incisional) is estimated to be around 0.08%-1% [1,^2^,4]. The majority of the appendix-containing hernia reported have occurred in groin, with inguinal hernia being the most common [1,4].

Appendicitis occurring in the incisional hernia is extremely rare [5]. The clinical presentation of an appendicitis in hernial sac varies, and do not present as a typical case of appendicitis, but rather with symptoms of incarcerated hernia [6]. Computed tomography (CT) plays a pivotal role in early diagnosis, however, there is paucity of data on efficacy of CT in diagnosis of herniated appendicitis.

In this report, we describe a case of appendicitis in the incisional hernia following renal transplantation.

## Case report

A 58-year-old male with clinical history of autosomal dominant polycystic kidney disease who previously underwent left total nephrectomy (in 2003) and renal transplantation (in 2003, 18 years prior to presentation) presents to the emergency department with acute onset right abdominal pain that gradually worsened over the span of 5 days. On arrival, the patient was afebrile and vital signs were stable. Physical examination revealed tenderness to deep palpation in the right lower quadrant and right flank.

Initial laboratory workup was as follow: White Blood Cell Count 16.1 × 10^9^/L, Hemoglobin 16.9 g/dL, Hematocrit 49.7%, Platelet 454 10^9^/L, Red Blood Cell Count 6.22 million/mm^3^, International Normalized Ratio (INR) 1.2, serum sodium 134 mEq/L, serum potassium 3.7 mEq/L, serum chloride 104 mEq/L, serum creatinine 1.04 mg/dL, blood urea nitrogen 15 mg/dL, and serum Lactate 0.9 mmol/L. Urine analysis was clean, reported as negative for glucose, bilirubin, ketones, blood, leukocytes, and protein, occasional bacteria, pH of 6.0 and specific gravity of 1.008. Urine Culture was reported no growth for 5 days.

**Fig. 1:**
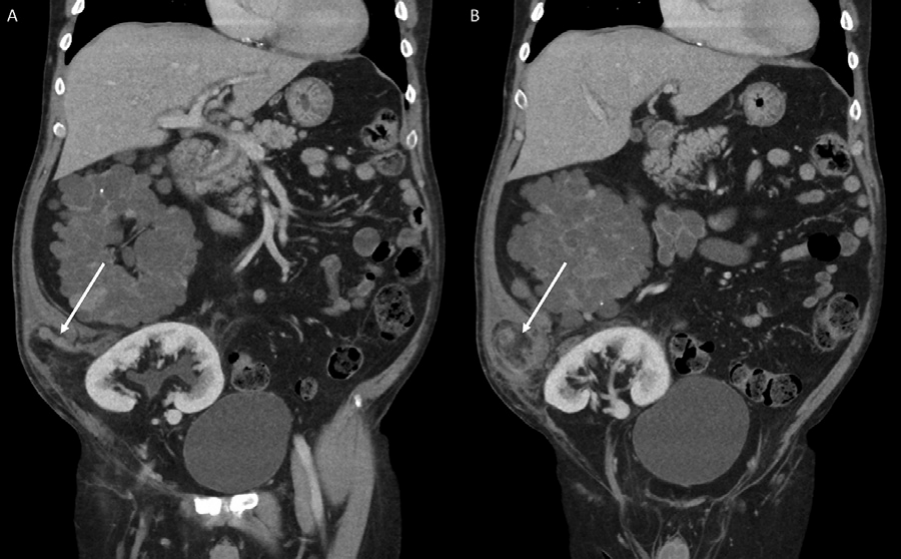
CT abdomen and pelvis with intravenous contrast administration, coronal view (Figs. A-B) demonstrates a wide neck incisional hernia in the right lower quadrant containing a tubular structure (white arrow) with associated diffuse edema in the hernial sac. The tubular structure demonstrates mild mucosal enhancement and wall thickening. Of note: The image also includes, right polycystic kidney and transplanted kidney in the right Iliac Fossa.

**Fig. 2:**
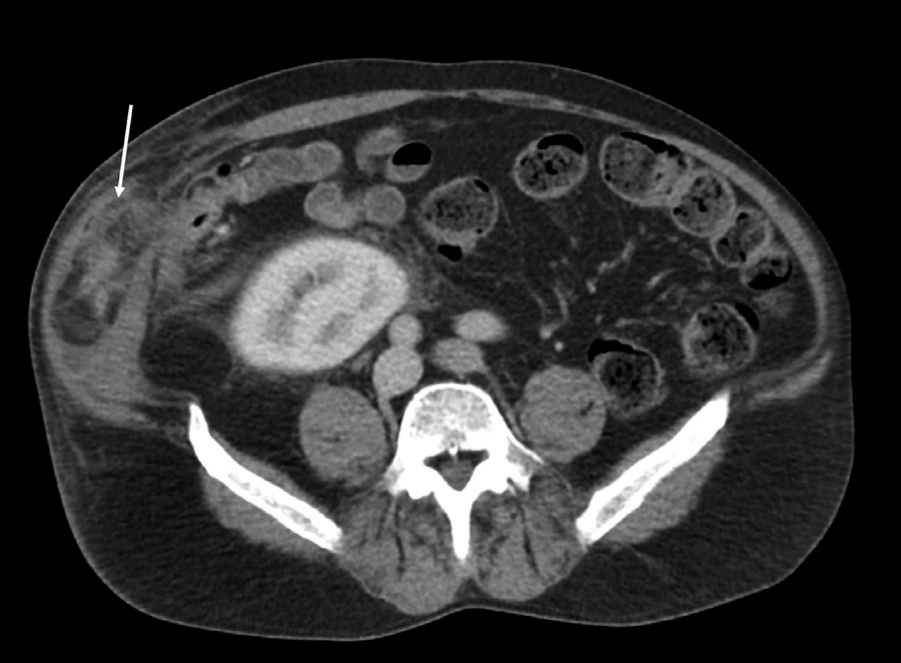
CT abdomen and pelvis with intravenous contrast administration, axial view demonstrates a wide necked incisional hernia in the right lower quadrant containing a tubular structure (white arrow) with associated diffuse edema in the hernial sac. Of note: The image also includes, transplanted kidney in the right Iliac Fossa.

**Fig. 3:**
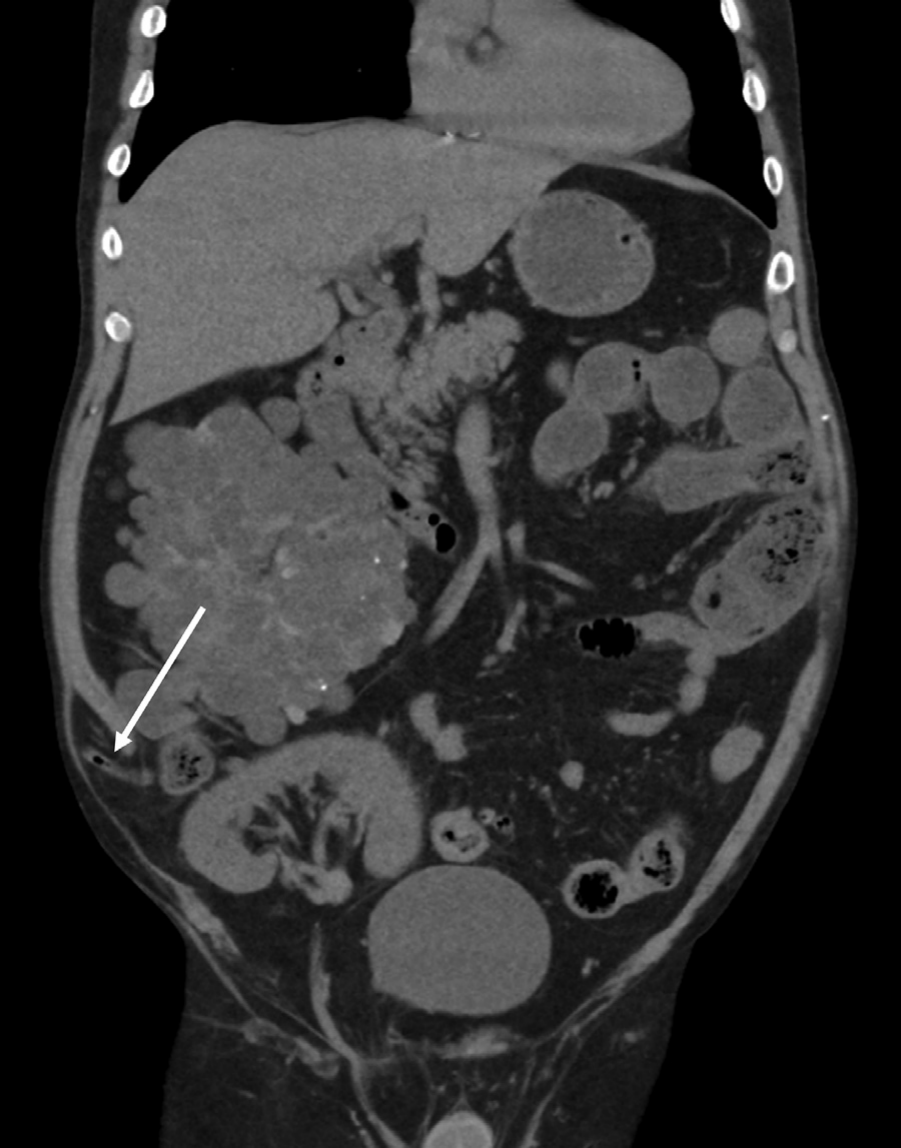
Prior CT abdomen and pelvis without intravenous contrast (performed 6 months prior to presentation) demonstrates presence of vermiform appendix (white arrow) in an incisional hernia sac. Of note: The image also includes, right polycystic kidney and transplanted kidney in right Iliac Fossa.

CT abdomen and pelvis with intravenous contrast was performed. A wide-neck right lower quadrant incisional hernia was noted, with stranding and inflammation within the hernial sac (Figs. 1 and 2). A distended tubular structure with mucosal enhancement was identified in the hernial sac, which was arising from the cecum. Based on imaging findings differential diagnoses included appendicitis, incarcerated hernia, or colonic diverticulitis. No intraperitoneal free air or pneumatosis was present. On comparison with prior CT abdomen and pelvis (6 months prior to presentation), a normal vermiform appendix was noted protruding into the incisional hernial sac (Fig. 3), making appendicitis as the leading differential for patients’ presentation.

Of note, other specific differential diagnosis to be considered in patients with renal transplantation include: perioperative acute tubular necrosis, renal allograft compartment syndrome, renal allograft torsion, acute or chronic renal transplant rejection, renal artery stenosis, renal vein thrombosis, arteriovenous fistula, perinephric fluid collection from infection or hematoma, renal artery pseudoaneurysm, urinary obstruction, graft pyelonephritis, opportunistic or community-acquired infection and donor-related malignancy [7].

Subsequently, exploratory laparotomy was performed which revealed a perforated appendicitis within the incisional hernial sac, confirming the diagnosis. Pathology reported as acute transmural appendicitis with perforation and ischemic changes. Viable proximal and distal mucosal margins and uninvolved by inflammatory process. Following surgery, the patient made an uneventful recovery.

## Discussion

Abdominal hernias may be congenital or acquired and are further classified based on the location, with inguinal hernia being the most common type [8]. Incisional hernia are relatively common with incidence ranging from 2%-11% after major abdominal surgery [9]. Asymptomatic incisional hernia is usually associated with higher morbidity and usually requires additional surgical intervention, and are typically associated with high recurrence rates (20%-46%). Further, the incidence of incisional hernia varies with the site of surgical incision. Midline incision is associated with higher rate of incisional hernia as compared to transverse, paramedian or oblique incision [10]. Conventionally, Pfannenstiel incision, used with gynecological procedures has the lowest incidence of incisional site hernia [10]. A systemic review in patients with renal transplant, revealed incisional hernia incidence of 1.1%-7% (mean 3.2%), significantly lower as compared to patients with midline laparotomy [11].

The presence of the appendix in the hernial sac is rare and in literature it is reported as low as 0.51% (out of 1950 patients with appendicitis) and has male predominance [12]. Historically, appendiceal hernia have been reported in inguinal hernias (referred as Amyand hernia), femoral hernias (referred as De Garengeots hernia), obturator hernias and incisional hernia [2]. The pathophysiology mechanism of dislocation of the appendix in the hernial sac is not fully understood. Proposed mechanism includes embryonal malrotation of the intestine, atypical position of the appendix or hypermobility of the cecum [13]. Dittmar et al reports a case of adherent appendix vermiform within an incisional hernia after renal transplantation mimicking acute appendicitis [14].

Appendicitis, inflammation of the vermiform appendix is a very common condition and major cause of abdominal surgery in young patients. It typically presents with periumbilical pain, right iliac fossa pain, fever and nausea-vomiting. The pathophysiology mechanism for appendicitis includes obstruction of the appendiceal lumen resulting in venous congestion, ischemia and/or necrosis and fluid accumulation [1]. CT is very sensitive in detecting appendicitis and is typically the first line imaging modality of choice in patients with suspected appendicitis. Ultrasound may be considered in patients with contraindication to CT, such as in pregnancy and pediatric age group. Typical CT findings include dilated appendix with a distended lumen (measuring greater than 6 mm in diameter), wall thickening and enhancement, peri-appendiceal inflammation, and occasionally an appendicolith [15].

Appendicitis within the hernia sac have a variable presentation and do not follow typical manifestations of acute appendicitis. Reported cases in the literature demonstrates similar clinical presentation to incarcerated hernia [6]. On imaging, the appendix appears as a blind-ending tubular structure that arises from the cecum and extends into the hernia sac. Findings suggestive of appendicitis includes dilation of the lumen, wall enhancement, wall thickening and peri-appendiceal fat stranding [15].

The management of non-inflamed appendix in the hernia sac remains controversial. A few reports have advocated surgical removal of any appendix encountered within an incisional hernia, even in absence of clinical and radiologic evidence of acute appendicitis [16,17]. Whereas, some report advocate against surgical intervention in non-inflamed appendix to avoid complications and risk of mesh infection [6].

Although appendicitis in the incisional hernia is a rare phenomenon, clinicians and radiologists should consider this diagnosis to prevent complications from delayed surgical treatment. CT might be helpful in early diagnosis.

## Patient consent

Authors received waiver of informed consent for this case report, since no identifiable information (PHI) was shared.
